# Polish validation of Brace Questionnaire

**DOI:** 10.1007/s00586-012-2188-0

**Published:** 2012-02-15

**Authors:** Edyta Kinel, Tomasz Kotwicki, Anna Podolska, Marianna Białek, Wanda Stryła

**Affiliations:** 1 Department of Rehabilitation, University of Medical Sciences, ul. 28 Czerwca 1956 135/147, 61-545 Poznan, Poland; 2 Spine Disorders Unit, Department of Paediatric Orthopaedics and Traumatology, University of Medical Sciences, ul. 28 Czerwca 1956 135/147, 61-545 Poznan, Poland; 3 FITS Center, Jawor, Poland

**Keywords:** Idiopathic scoliosis, Quality of life, Cultural adaptation, Brace Questionnaire

## Abstract

**Purpose:**

The aim of the study was to undertake the process of cultural adaptation of the Brace Questionnaire (BrQ) into Polish.

**Methods:**

The BrQ is an instrument for measuring the quality of life of scoliotic adolescents who are being treated conservatively with wearing a corrective brace. The BrQ consists of 34 Likert-scale items related to eight domains. The translation from the original Greek into Polish was performed. The process of cultural adaptation of the questionnaire was in accordance with the guidelines of the International Quality of Life Assessment Project. It involved 35 adolescents, aged between 10.0 and 16.0 years, all with adolescent idiopathic scoliosis with mean Cobb angle of 35.1 ± 10.6 degrees, and all wearing the same kind of brace (Chêneau orthosis) for more than 3 months. Statistical analysis calculated the reliability (internal consistency), floor and ceiling effects of the BrQ.

**Results:**

The internal consistency was satisfactory; Cronbach’s alpha coefficient was 0.94. There was no floor or ceiling effects.

**Conclusions:**

Polish version of the BrQ is reliable and can be used in adolescents with idiopathic scoliosis wearing the brace to assess their quality of life.

## Introduction

Adolescent idiopathic scoliosis (AIS) is a complex and progressive condition, which can affect patients’ quality of life (QoL). Changing the QoL should be taken into account when evaluating treatment results of AIS patients [1].

There are a few QoL questionnaires dedicated for patients with AIS: SRS-22, Scoliosis Quality of Life Index (SQLI) and SF-36 seem to be the most diffused [2, 3]. They are applied to patients who have already completed their treatment.

Two questionnaires for monitoring the level of stress in patients currently being managed conservatively for progressive scoliosis have been constructed: they are designated to assess the stress induced by the deformity (Bad Sobernheim Stress Questionnaire—Deformity, BSSQ-Deformity), as well as the stress induced by the treatment with a brace (Bad Sobernheim Stress Questionnaire—Brace, BSSQ-Brace) [4, 5]. These questionnaires do not assess the overall QoL.

In 2006 Vasiliadis et al. [6] proposed the Brace Questionnaire (BrQ), an instrument for measuring the QoL of scoliotic adolescents who are being treated conservatively with wearing a corrective brace. This tool was developed and validated in the Greek language. Being familiar with its English version (not validated), we have found this questionnaire to be potentially helpful for monitoring the QoL of our patients.

The aim of the study was to carry on the process of cultural adaptation of the BrQ into Polish.

## Subjects and methods

Brace Questionnaire is a 34 Likert scale items questionnaire, and consists of eight specific domains; they are: (a) general health perception, (b) physical functioning, (c) emotional functioning, (d) self-esteem and aesthetics, (e) vitality, (f) school activity, (g) bodily pain and (h) social functioning [6]. The questionnaire was designed to be self-administrated and developmentally appropriate for ages 9–18 years. Scoring of the BrQ was planned as follows: for items 4, 5, 6, 12, 14, 15, 16, 17 “Always” receives a score of 5, “Most of the time” receives a score of 4, “Sometimes” receives a score of 3, “Almost never” receives a score 2 and “Never” receives a score of 1. For items 1, 2, 3, 7, 8, 9, 10, 13, 18, 19, 20, 21, 22, 23, 24, 25, 26, 27, 28, 29, 30, 31, 32, 33, 34 “Always” receives a score of 1, “Most of the time” receives a score of 2, “Sometimes” receives a score of 3, “Almost never” receives a score of 4 and “Never” receives a score of 5. Next, each item score is multiplied by 20 and the total score is divided by 34. Thus, the minimum score of the questionnaire is 20 and the maximum is 100. Higher scores are better quality of life. A subscale score can be calculated for each of the eight domains by dividing the total score of each dimension by the number of its items [6].

## Adaptation process

The process of cross-cultural adaptation of the BrQ was performed in accordance with the guidelines set up by International Quality of Life Assessment (IQOLA) [7].

In the first stage, two independent translators converted the original Greek text into Polish. One of the translators, who had a medical background, was instructed on the whole process of adaptation. The other translator had no medical background and received no information on the project. Second stage consisted of comparison of the original and two translated versions. During that stage, the two translators and the authors identified differences in translations and produced a combined version. In the third stage—the so-called reversed translation—two independent translators, who were native in Greek, translated the Polish version into the language of the original document (Greek). The translators were not familiar with the original version. The objective of this stage was to assure equivalence of the two versions and to identify possible mistranslations. At the last fourth stage, a commission composed of a specialist in orthopaedics, translators, a statistician and a psychologist reviewed the translations. As a result of consensus, the so-called pre-final version was drafted.

Thirty-five patients with idiopathic scoliosis were enrolled for the assessment using the Polish version of BrQ, twice within one-week interval. The duration of the first attempt to complete the questionnaire was measured. The sample included 28 girls and 7 boys. All patients were treated with the same kind of brace (Chêneau orthosis) and by the same specialist in orthopaedics (second author). All patients and parents gave their informed consent prior to their inclusion in the study. The following inclusion criteria were applied: (1) patients at the age of 9–18 years, (2) who have been wearing the brace for at least 3 months for at least 12 h per day, (3) with Cobb angle between 20 and 45°, (4) having thoracic, thoracolumbar or lumbar scoliosis. Mean age of the patients at the time of completing the questionnaire was 14.0 years (±1.5 years), for details see Table 1. Patients have been wearing the brace for an average duration of 17.9 months (±11.7 months). Patients have been wearing the brace for 17.0 h per day (±5.2 h). Among the patients, 22.9% had thoracic scoliosis, 62.9% thoracolumbar scoliosis and 14.2% lumbar scoliosis. 25.7% of patients had left curve pattern and 74.3% of patients had right curve pattern.

**Table 1:** Description of the study subjects

	Mean (SD)
Age (years)	14.0 (1.5)
Body weight (kg)	48.0 (8.9)
Height (cm)	162.5 (8.4)
Cobb angle (degrees)	35.1 (10.6)
Angle of trunk rotation^a^ (degrees)	7.0 (2.9)

^a^Angle of trunk rotation as measured with Bunnell scoliometer

## Statistical analysis

Statistical analysis was performed using Statistica 9.1 software. Shapiro–Wilk test for normality did not identify the data to be normally distributed; therefore, non-parametric tests were used*.* Two levels of analysis were applied. Firstly, descriptive statistics was used to calculate mean scores and standard deviations for a given question and a domain. The second level was comparative, concerning reliability and validity.

Reliability. The two most important properties of reliability are consistency and stability. Internal consistency was assessed using Cronbach’s alpha coefficient. Test–retest design was used to measure temporal stability of the questionnaire with Kendall’s tau (r) coefficient. To reduce the memory effect, there was a 7-day period between tests [1].

Validity. The BrQ was assessed for item convergent validity (item-scale correlation should be ≥0.4), floor and ceiling effects. The distribution of results indicates the number (percentage) of patients with minimum score (floor effect) and the number (percentage) of patients with maximum score (ceiling effect) [6].

## Ethical considerations

Polish adaptation of BrQ as a research project has been approved by the Bioethical Commission at the University (decision number 541/11).

## Results

Average, lowest, highest scores and 95% confidence interval obtained using the BrQ are presented in Table 2. The mean score for the BrQ was 77.1 points (±12.2 points) in the first test and 76.5 points (±12.1 points) in the second test. The mean duration of completing the questionnaire was 7.9 min (±1.36 min).

**Table 2:** Distribution of mean, minimal and maximal scores, 95% confidence interval of Polish BrQ

Questionnaire	*N*	Min	Max	Mean	95% confidence interval		Standard deviation
					From	To	
BrQ first trial	35	52	92	77.1	70.7	79.1	12.2
BrQ second trial	35	52	91	76.5	71.3	79.6	12.1

Value of Cronbach’s alpha and Kendall’s tau (r) coefficient of the Polish version of the BrQ assessed with the use of test–retest method in comparison with Greek results are presented in Table 3.

**Table 3:** The value of Cronbach’s alpha and Kendall’s tau coefficients

Questionnaire	Polish version		Greek version (original)	
	Cronbach alpha	Kendall tau (r)	Cronbach alpha	Kendall tau (r)
BrQ	0.94	0.82	0.82	–

Mean, standard deviation, floor and ceiling effects for each BrQ question are presented in Table 4, while the values for each BrQ domain are presented in Table 5. Mean values for individual BrQ questions ranged from 1.0 (question 34) to 5.0 (questions 5, 6, 20, 22, 28 and 31).

**Table 4:** Mean, standard deviation, floor and ceiling effects for each BrQ question

BrQ	Mean	Standard deviation	Number (%)	
			Floor effect	Ceiling effect
Question 1	4.0	0.8	0 (0.0)	11 (31.4)
Question 2	3.5	1.0	0 (0.0)	7 (20.0)
Question 3	3.5	1.2	1 (2.9)	6 (17.1)
Question 4	3.0	1.2	4 (11.4)	4 (11.4)
Question 5	5.0	0.5	0 (0.0)	32 (91.4)
Question 6	5.0	0.1	0 (0.0)	33 (94.3)
Question 7	4.0	1.1	0 (0.0)	9 (25.7)
Question 8	4.0	1.2	2 (5.7)	8 (22.9)
Question 9	4.0	1.0	1 (2.9)	7 (20.0)
Question 10	4.0	0.9	0 (0.0)	13 (37.1)
Question 11	4.0	1.0	0 (0.0)	12 (34.3)
Question 12	3.0	1.2	6 (17.4)	0 (0.0)
Question 13	2.5	1.4	5 (14.3)	5 (14.3)
Question 14	4.0	1.0	0 (0.0)	11 (31.4)
Question 15	3.5	1.0	0 (0.0)	6 (17.4)
Question 16	3.0	1.0	2 (5.7)	1 (2.9)
Question 17	3.5	0.9	1 (2.9)	2 (5.7)
Question 18	3.5	1.2	3 (8.6)	3 (8.6)
Question 19	4.5	1.0	0 (0.0)	16 (45.7)
Question 20	5.0	0.8	0 (0.0)	31 (88.6)
Question 21	4.5	1.0	0 (0.0)	17 (48.6)
Question 22	5.0	0.6	0 (0.0)	26 (74.3)
Question 23	4.5	0.9	0 (0.0)	16 (45.7)
Question 24	4.0	1.0	0 (0.0)	9 (25.7)
Question 25	4.0	1.0	0 (0.0)	10 (28.6)
Question 26	4.5	0.9	0 (0.0)	15 (42.9)
Question 27	3.5	1.4	3 (8.6)	11 (31.4)
Question 28	5.0	1.1	2 (5.7)	18 (51.4)
Question 29	3.0	1.1	3 (8.6)	2 (5.7)
Question 30	3.5	1.4	2 (5.7)	9 (25.7)
Question 31	5.0	0.7	0 (0.0)	24 (68.6)
Question 32	4.5	1.2	2 (5.7)	15 (42.9)
Question 33	4.0	1.1	1 (2.9)	14 (40.0)
Question 34	1.0	1.0	22 (62.9)	1 (2.9)

**Table 5:** Mean and standard deviation for each BrQ domain

BrQ Domain	Number of items	Median	Standard deviation
General health perception	2	7.5	1.5
Physical functioning	7	27.5	4.3
Emotional functioning	5	17.5	4.2
Self esteem and aesthetics	2	7.0	2.0
Vitality	2	7.0	1.7
School activity	3	14.0	2.2
Bodily pain	6	25.5	4.4
Social functioning	7	25.5	5.0

Item convergent validity, Cronbach’s alpha, floor and ceiling effects for each BrQ domain are presented in Table 6. There were no floor or ceiling effects when completing the questionnaire for the first and the second time.

**Table 6:** Item convergent validity, Cronbach’s alpha and floor and ceiling effects for each BrQ domain

BrQ domain	Number of items	Item convergent validity (%)	Cronbach’s alpha	Floor effect (%)	Ceiling effect (%)
General health perception	2	100	0.51	0 (0)	3 (8)
Physical functioning	7	30	0.74	0 (0)	0 (0)
Emotional functioning	5	100	0.82	0 (0)	0 (0)
Self esteem and aesthetics	2	100	0.91	0 (0)	1 (3)
Vitality	2	0	0.52	0 (0)	0 (0)
School activity	3	100	0.71	0 (0)	0 (0)
Bodily pain	6	70	0.82	0 (0)	4 (11)
Social functioning	7	40	0.77	0 (0)	0 (0)

## Discussion

### Statistical relevance

Cronbach’s alpha is considered to be a proper method for estimating reliability of multi-item scales, it provides an estimate of internal consistency that expresses both the number of items and their average correlation. Even though Cronbach’s alpha disregards other possible material sources of measurement error (e.g. temporal instability), these sources of measurement error usually have a minimal impact on the measure of reliability [3]. Cronbach’s alpha should be greater than 0.80 to prove good reliability [8]. The Polish version of the BrQ had a high value of Cronbach’s alpha coefficient (0.94), exceeding the minimum recommended value of 0.80 and indicating satisfactory internal consistency as a factor of satisfactory reliability of the BrQ. Cronbach’s alpha overall score achieved by Vasiliadis et al. [6] was 0.82. Preliminary validation of the Italian version of the BrQ questionnaire had the value of Cronbach’s alpha of 0.86, indicating satisfactory internal consistency [1].

Kendall’s tau (r) coefficient of the Polish version of the BrQ assessed with the use of test–retest method was 0.82. The criterion for item convergent validity (item-scale correlations ≥0.40) was fulfilled by the items related to general health perception, emotional functioning, self-esteem and aesthetics, school activity, bodily pain and social functioning. In the present study, the criterion for item convergent validity was not fulfilled by the items related to physical functioning and vitality. In the Italian validation, the test–retest reliability showed a good temporal stability (*r* = 0.88, *p* < 0.001) [1].

For the BrQ overall score, in the present study 0% of patients scored at floor and 0% scored at ceiling. Therefore, there were no floor or ceiling effects for the BrQ overall score. Vasiliadis et al. [6] reported similar results.

### Clinical relevance

Conservative treatment of scoliosis with a rigid brace can have a significant impact on patients’ wellbeing and negatively affect their QoL [1]. AIS can lead to multiple impairments, non-only of physical but also of psychosocial character [7, 9]. The effectiveness of the conservative scoliosis treatment has been demonstrated to be dependent on the patients’ treatment compliance [10, 11].

The level of stress during therapy is one of the factors determining compliance and can be assessed using the BSSQ questionnaire [12]. Weiss [4] reported brace treatment to be associated with higher level of stress and poor quality of life. Kotwicki et al. noticed that the BSSQ is helpful for determining the level of stress during scoliosis therapy. Misterska et al. [13] described Polish adaptation of the BSSQ. However, the BSSQ is not able to measure the influence of family, school environment or physical activity on patient’s QoL [11]. The BrQ is the first questionnaire specially developed and validated to measure the quality of life of adolescent currently being under conservative scoliosis treatment with a corrective brace [6].

According to Vasiliadis and Grivas, when assessing the effectiveness of conservative treatment of AIS, the health-related quality of life (HRQoL) variables are more important than radiographic results or pulmonary function tests [14]. Lee et al. emphasize growing interest in demonstrating the effect of treatments on the health-related quality of life of patients with idiopathic scoliosis [15].

According to Vasiliadis et al. [6], a specific instrument, such as the BrQ, has evident strengths by virtue of its increased sensitivity to the problems related to the brace itself. Aulisa et al. [1] emphasize that QoL monitoring should be routinely implemented during brace treatment, with type of bracing, gender, curve pattern and Cobb angle taken into account, to provide professional psychological support if needed. Our results indicate that the BrQ is an effective tool for evaluating QoL of patients with AIS being treated with a corrective brace.

## Conclusion

The BrQ takes less than 10 min to be completed and covers most of the aspects of life affected by the brace. Polish version of the BrQ is reliable and can be used in adolescents with idiopathic scoliosis wearing the brace to assess their quality of life.

## Appendix

Polish version of Brace Questionnaire

ANKIETA—GORSET ORTOPEDYCZNY

*Poniższa ankieta zawiera pytania dotyczącego tego, co myślisz o stanie swojego zdrowia i jak się czujesz. Nie jest to żaden test, w którym istnieją poprawne i błędne odpowiedzi.*

- *Przeczytaj uważnie każde pytanie.*
- *Wybierz odpowiedź, którą uważasz za właściwą i postaw X w odpowiednim kwadracie.*

**Table:**

*Przykład*	Nigdy	Rzadko	Czasami	Często	Zawsze
W ubiegłym tygodniu miałam/-em ochotę do nauki	□	□	□	*x*	□

Prosimy, o podanie nam informacji dotyczących ciebie:

Jesteś: □ dziewczyną □ chłopcem Wiek: ……… lat

Data………………………………………….

**Table:**

*W ostatnich trzech miesiącach…*	Nigdy	Rzadko	Czasami	Często	Zawsze
1. Gorset sprawiał, że czułaś/-eś się chora/-y	□	□	□	□	□
2. Obawiasz się, że twoja skolioza/skrzywienie kręgosłupa powiększa się	□	□	□	□	□
3. Z powodu noszenia gorsetu męczyłaś/-eś się przy chodzeniu	□	□	□	□	□
4. Mogłaś/-eś biegać w gorsecie	□	□	□	□	□
5. Zakładałaś/-eś gorset samodzielnie	□	□	□	□	□
6. Samodzielnie zdejmowałaś/-eś gorset	□	□	□	□	□
7. Nie mogłaś/-eś wygodnie jeść, ponieważ nosiłaś/-eś gorset	□	□	□	□	□
8. Nie spałaś/-eś dobrze ze względu na gorset	□	□	□	□	□
9. Nie mogłaś/-eś swobodnie oddychać ze względu na gorset	□	□	□	□	□
10. Gorset sprawiał, że czułaś/-eś się nerwowy/-a	□	□	□	□	□
11. Z powodu noszenia gorsetu czułaś/-eś się smutna/-y	□	□	□	□	□
12. Czułaś/-eś się szczęśliwa/-y	□	□	□	□	□
13. Uważasz, że twoje życie byłoby lepsze bez noszenia gorsetu	□	□	□	□	□
14. Uważasz, że terapia z zastosowaniem gorsetu była dla ciebie korzystna	□	□	□	□	□

*W ubiegłym miesiącu…*	Nigdy	Rzadko	Czasami	Często	Zawsze
15. Byłaś/-eś z siebie dumny/-a	□	□	□	□	□
16. Byłaś/-eś z siebie zadowolona/-y	□	□	□	□	□
17. Czułaś/-eś się silna/-y i pełna/-en energii	□	□	□	□	□
18. Z powodu noszenia gorsetu czułaś/-eś się zmęczona/-y i wyczerpana/-y	□	□	□	□	□
19. Z powodu noszenia gorsetu miałaś/-eś trudności w odrabianiu lekcji i z nauką	□	□	□	□	□
20. Z powodu noszenia gorsetu opuszczałaś/-eś zajęcia szkolne	□	□	□	□	□
21. Byłaś/-eś roztargniona/-y na lekcjach i w klasie	□	□	□	□	□
22. Brałaś/-eś leki, ponieważ odczuwałaś/-eś ból	□	□	□	□	□
23. Odczuwałaś/-eś ból w nocy	□	□	□	□	□
24. Odczuwałaś/-eś ból podczas chodzenia	□	□	□	□	□
25. Odczuwałaś/-eś ból podczas siedzenia	□	□	□	□	□
26. Odczuwałaś/-eś ból przy chodzeniu po schodach	□	□	□	□	□
27. Z powodu noszenia gorsetu czułaś/-eś cierpnięcie rąk lub nóg	□	□	□	□	□
28. Gorset utrudniał ci spotkania z koleżankami/kolegami	□	□	□	□	□
29. Z powodu twoich problemów z plecami twoje koleżanki/koledzy współczuli ci,	□	□	□	□	□
30. Z powodu noszenia gorsetu czułaś/-eś się inna/-y niż twoje koleżanki/koledzy,	□	□	□	□	□
31. Z powodu noszenia gorsetu miałaś/-eś problemy ze swoją rodziną	□	□	□	□	□
32. Uważasz, że twoje relacje z rodziną i koleżankami/kolegami byłyby lepsze, gdybyś nie nosiła/-ł gorsetu	□	□	□	□	□
33. Pozostawałaś/-eś w domu, ponieważ wstydziłaś/-eś się gorsetu	□	□	□	□	□
34. Z powodu noszenia gorsetu zakładałaś/-eś specjalne ubrania (ukrywające gorset)	□	□	□	□	□
